# Modulation of endogenous **β**-tubulin isotype expression as a result of human **β**_III_ cDNA transfection into prostate carcinoma cells

**DOI:** 10.1054/bjoc.2001.1956

**Published:** 2001-09

**Authors:** S Ranganathan, R A McCauley, D W Dexter, G R Hudes

**Affiliations:** Penn State College of Medicine, Dept of Pharmacology, 500 University Drive, Hershey, PA, 17033, USA; Department of Medical Oncology, Fox Chase Cancer Center, 7701 Burholme Avenue, Philadelphia, PA 19111

**Keywords:** tubulin, isotype, antimicrotubule agents, drug resistance, transfection

## Abstract

Increases of individual β tubulin isotypes in antimicrotubule drug resistant cell lines have been reported by several laboratories. We have previously described elevations in β_III_ and β_IVa_ isotypes in estramustine and paclitaxel resistant human prostate carcinoma cells. To investigate further the function of β tubulin isotypes in antimicrotubule drug response, human prostate carcinoma cells that normally have very low to undetectable levels of β_III_ were stably transfected with β_III_ cDNA in pZeoSV system. An 18 bp haemagglutinin (HA) epitope tag was added at the 3′ end prior to cloning into the vector. Cells were transfected with pZeoSV or pZeoSV-β_III_ plasmids and selected in the presence of Zeocin. Immunofluorescent staining of the transfectant cells have shown significant expression and incorporation of HA-tagged β_III_ tubulin into cellular microtubules. Quantitation of Western blots revealed the HA-tagged β_III_ levels to be approximately 7-fold higher than the vector control cells. RT-PCR analysis confirmed the increase at the transcript level and also revealed a collateral increase of β_II_ and β_IVb_ transcripts. Cell viability assays indicated that sensitivity of β_III_ transfected cells to various antimicrotubule agents was similar to vector transfected cells: IC50 values for estramustine, paclitaxel, colchicine and vinblastine were 4 μM, 4 nM, 22 nM and 2 nM, respectively for both cell lines. Thus, overexpression of β_III_ isotype in human prostate carcinoma cells by stable transfection failed to confer antimicrotubule drug resistance to these cells. Counterregulatory increases of endogenous β_II_ and β_IVb_ tubulin isotypes in these β_III_ transfected cells may be a compensatory mechanism used by the cells to overcome the effects of elevated β_III_ levels on the cellular microtubules. These results highlight the difficulty in isolating the contribution of single tubulin isotypes in drug response studies. © 2001 Cancer Research Campaign http://www.bjcancer.com


*British Journal of Cancer* (2001) **85**(5), 735–740
© 2001 Cancer Research Campaign
doi: 10.1054/ bjoc.2001.1956, available online at http://www.idealibrary.com on IDEAL

http://www.bjcancer.com

### Modulation of endogenous β-tubulin isotype expression as a result of human $\beta_{III}$ cDNA transfection into prostate carcinoma cells

**S Ranganathan, RA McCauley, DW Dexter[1] and GR Hudes**

Department of Medical Oncology, Fox Chase Cancer Center, 7701 Burholme Avenue, Philadelphia, PA 19111; and [1]Penn State College of Medicine, Dept of Pharmacology, 500 University Drive, Hershey, PA 17033, USA

**Summary** Increases of individual β tubulin isotypes in antimicrotubule drug resistant cell lines have been reported by several laboratories. We have previously described elevations in $\beta_{III}$ and $\beta_{IVa}$ isotypes in estramustine and paclitaxel resistant human prostate carcinoma cells. To investigate further the function of β tubulin isotypes in antimicrotubule drug response, human prostate carcinoma cells that normally have very low to undetectable levels of $\beta_{III}$ were stably transfected with $\beta_{III}$ cDNA in pZeoSV system. An 18 bp haemagglutinin (HA) epitope tag was added at the 3′ end prior to cloning into the vector. Cells were transfected with pZeoSV or pZeoSV-$\beta_{III}$ plasmids and selected in the presence of Zeocin. Immunofluorescent staining of the transfectant cells have shown significant expression and incorporation of HA-tagged $\beta_{III}$ tubulin into cellular microtubules. Quantitation of Western blots revealed the HA-tagged $\beta_{III}$ levels to be approximately 7-fold higher than the vector control cells. RT-PCR analysis confirmed the increase at the transcript level and also revealed a collateral increase of $\beta_{II}$ and $\beta_{IVb}$ transcripts. Cell viability assays indicated that sensitivity of $\beta_{III}$ transfected cells to various antimicrotubule agents was similar to vector transfected cells: IC50 values for estramustine, paclitaxel, colchicine and vinblastine were 4 μM, 4 nM, 22 nM and 2 nM, respectively for both cell lines. Thus, overexpression of $\beta_{III}$ isotype in human prostate carcinoma cells by stable transfection failed to confer antimicrotubule drug resistance to these cells. Counterregulatory increases of endogenous $\beta_{II}$ and $\beta_{IVb}$ tubulin isotypes in these $\beta_{III}$ transfected cells may be a compensatory mechanism used by the cells to overcome the effects of elevated $\beta_{III}$ levels on the cellular microtubules. These results highlight the difficulty in isolating the contribution of single tubulin isotypes in drug response studies. © 2001 Cancer Research Campaign http://www.bjcancer.com

**Keywords**: tubulin; isotype; antimicrotubule agents; drug resistance; transfection

In the past several years many laboratories have studied the significance of α and β tubulin proteins in antimicrotubule drug resistance. These studies have extended to several antimicrotubule agents in various carcinoma cell lines. Early studies showed mutations and alterations in the steady state soluble and polymerized α and β tubulin fractions in drug resistant cell lines (Schibler and Cabral, 1986; Cabral et al, 1986; Minotti et al, 1991). Although the existence of different α and β tubulin isotypes and their tissue specific expression was reported in the past, only recently demonstrated were the effects of individual β tubulin isotypes on overall microtubule functions including assembly, dynamics, drug sensitivity and drug binding. Banerjee et al (1990) have shown that $\beta_{III}$ isotype depleted tubulin assembles into microtubules at a faster rate than unfractioned tubulin. These microtubules are also more sensitive to paclitaxel-induced assembly compared to unfractioned tubulin (Lu and Luduena, 1993). Subsequent studies revealed alterations in the expression of specific β tubulin isotypes as result of antimicrotubule drug resistance (Haber et al, 1995; Ranganathan et al, 1996; 1998a; 1998b; Kavallaris et al, 1997; Kavallaris et al, 1999). In addition, combination studies have shown that cyclosporin A enhances paclitaxel efficacy in lung carcinoma cell lines by modulating β tubulin isotype composition (Ross and Antoniono, 1999). Recently, Kavallaris et al, (1999) have shown that antisense oligonucleotides to $\beta_{III}$ isotype sensitized the drug resistant cells to paclitaxel. In addition, mutations in $\beta_{I}$ isotype were reported in paclitaxel resistant human ovarian carcinoma cells (Giannakakou et al, 1997), breast carcinoma cells (Wiesen and Horwitz, 2000) and non-small-cell lung cancer patients (Monzo et al, 1999) with tumours that were unresponsive to paclitaxel therapy.

Antimicrotubule drug resistance associated changes in tubulin isotypes prompted several investigators to determine the contribution of individual isotypes by transfecting cells and examining their antimicrotubule drug response. Wu et al (1998) have shown that transfection of $\beta_{IVa}$ isotype into human leukemic cell line failed to confer resistance to paclitaxel. Blade et al (1999) have over-expressed rodent $\beta_{I}$, $\beta_{II}$ or $\beta_{IVb}$ isotypes in CHO cells and shown that these isotypes do not confer resistance to paclitaxel. Our previous work demonstrated increases in $\beta_{III}$ and $\beta_{IVa}$ isotypes in human prostate carcinoma cells that were made resistant to estramustine or paclitaxel (Ranganathan et al, 1996, 1998a). In addition, acute exposures to these agents resulted in elevations of $\beta_{III}$ levels. To further understand the function of $\beta_{III}$ isotype in antimicrotubule drug response, parental DU145 human carcinoma cells were transfected with the human $\beta_{III}$ cDNA. The results presented herein indicate that regulation of tubulin in cells is complex and that attempts to increase levels of a single isotype may lead to compensatory changes in the expression of other isotypes.

*Received 30 August 2000*
*Revised 30 May 2001*
*Accepted 31 May 2001*

*Correspondence to:* S Ranganathan

### MATERIALS AND METHODS

### Construction of expression vectors and selection of transfectants

Human $\beta_{III}$ cDNA that was previously cloned in our laboratory (Ranganathan et al, 1998b) was inserted into the HindIII and XhoI sites of pZeoSV vector (Invitrogen, Carlsbad, CA). An 18 bp haemagglutinin tag (HA) was added at the C-terminus to distinguish the vector driven $\beta_{III}$ protein from the endogenous protein. Human prostate carcinoma (DU145) cells were transfected with pZeoSV or pZeo$\beta_{III}$ DNA by using superfect (Qiagen, Santa Clarita, CA) according to manufacturer's protocol. Cells were placed in drug selection medium containing 100 μg/mL Zeocin and clones were selected.

### Immunofluorescent staining of human $\beta_{III}$-transfectant cells

$\beta_{III}$-transfectant and vector-transfectant cells were plated onto glass coverslips and allowed to attach. Cells were fixed in 4% paraformaldehyde and stained in the following manner. Cells were treated with 3% BSA in phosphate buffered saline (PBS) for 30 min to block for non-specific binding of the antibodies. Cells were then stained with the following antibodies diluted in 1% BSA/PBS for 90 min at 37˚C: pan α tubulin antibodies, pan β tubulin antibodies (1:200, Sigma Chemical Co., St. Louis, MO), $\beta_{II}$, $\beta_{III}$ and $\beta_{IV}$ isotype-specific antibodies (1:100, Biogenex, San Ramon, CA) and haemagglutinin antibodies (1:500, BabCo, Richmond, CA). Cells were stained with rhodamine-red-x conjugated antimouse secondary antibodies (1:800, Jacksonville Immunoresearch,) for 45 min at 37˚C, mounted onto slides, and examined. Images were captured with 12 bit cooled CCD (Quantix, Photometrics, Tuscon, AZ) and average pixel intensity values were quantitated and analysed by using Isee software (Inovision Corp., Raleigh/Durham, NC). At least 200 cells were quantited per each antibody stain and the average pixel intensities were calculated.

### Western blot analysis of proteins from $\beta_{III}$-transfectant and vector-transfectant cells

Cytosolic fractions were prepared from the cells and Western blots were analysed as described previously (Ranganathan et al, 1996). Briefly, cells were lysed for 10 min at 4˚C in PBS containing 1% Triton X100, 0.1% SDS, 0.5% Sodium deoxycholate and protease inhibitors, followed by centrifugation at 10 000 × *g* for 10 min. Protein concentrations of the supernatants were estimated by the Biorad method (Hercules, CA). Proteins were fractionated by 8% polyacrylamide gel electrophoresis and transferred onto PVDF membranes (Millipore, Bedford, MA). Western blots were probed with antibodies against HA epitope (1:500) and $\beta_{III}$ tubulin isotype (1:200) and developed using ECL plus chemiluminiscence detection system (Amersham Pharmacia Biotech, Piscataway, NJ). Results were quantitated by densitometric scanning of the films.

### RT-PCR analysis of β tubulin isotype transcripts from pZeoSV and pZeo$\beta_{III}$-transfectant cells

RNA was isolated from both cell lines using RNAeasy kit from Qiagen Inc (Chadsworth, CA). β tubulin isotype-specific primers for the $\beta_I$, $\beta_{II}$, $\beta_{III}$, $\beta_{IVa}$ and $\beta_{IVb}$ isotypes and the PCR conditions were described in detail previously (Ranganathan et al, 1996). In addition, vector-specific and HA epitope specific primers were chosen using the primer detective program (Clontech, Palo Alto, CA) and synthesized by the DNA core facility at Fox Chase Cancer Center. PCR products were analysed by agarose gel electrophoresis and quantitated by densitometric scanning.

### Cytotoxicity assays

ZeoSV and Zeo$\beta_{III}$ transfectant cell lines were plated onto 96-well cell culture plates and allowed to attach. Estramustine was a gift from Kabi Pharmacia, Lund, Sweden (now Pharmacia, Bridgewater, NJ). Paclitaxel, vinblastine and colchicine were purchased from Sigma Chemical Co. Cells were exposed to various concentrations of the drugs for 48 h, fixed and stained with sulforhodamine B, as previously described (Ranganathan et al, 1996). Absorbance was measured at 560 nm and cell survivals in both cell lines were determined.

### RESULTS

### Expression of $\beta_{III}$ tubulin isotype by transfection

When the cells were stained with antibodies against HA epitope, $\beta_{III}$ transfected cells showed significant staining for the HA epitope in the microtubules, indicating $\beta_{III}$ protein was derived from the pZeo$\beta_{III}$ DNA and was incorporated into cellular microtubules (Figure 1D). By contrast, staining for the vector-transfected cells was similar to background level of staining (Figure 1C). When the cells were stained with the antibodies against $\beta_{III}$ isotype, a 2.4-fold increase in staining was seen in the $\beta_{III}$ transfectant cells (Figure 1B) compared to the vector transfected cells (Figure 1A). Cells were also stained with antibodies against pan α and pan β tubulin proteins to determine if the over-expression of $\beta_{III}$ isotype had any affect on the overall β and α tubulin levels (Figure 1 E–H). As shown in the figure, levels of β tubulin levels did not change significantly from vector-transfected cells (Figure 1E) to $\beta_{III}$ transfected cells (Figure 1F). There was a 1.8-fold increase of overall α tubulin levels in $\beta_{III}$ transfected cells (Figure 1H) compared to vector transfectant cells (Figure 1G). Quantiation of immunofluorescent staining was done as described in the Methods section and the results are shown in Table 1.

Western blot analysis of the proteins from the transfectant cell lines by use of HA antibodies has confirmed the increase seen by immunofluorescence (Figure 2). Quantitation of the Western blot by NIH image analysis software revealed a 7-fold increase in the HA signal. There were no differences in the overall β tubulin and β tubulin levels between the vector transfected and $\beta_{III}$ transfected cell lines.

**Table 1** Quantitation of immunofluorescent images of $\beta_{III}$ transfected and vector transfected cells stained with antibodies. Cells were stained, images were captured and average pixel intensities were quantitated as described in the Methods section

| Antibody used | Vector-transfectants | $\beta_{III}$ transfectants |
|---|---|---|
| $\beta_{III}$ tubulin | 339 ± 24 | 828 ± 33 |
| HA-tag | 89 ± 17 | 407 ± 55 |
| Pan β-tubulin | 637 ± 67 | 866 ± 33 |
| Pan α-tubulin | 610 ± 42 | 1120 ± 27 |

 *© 2001 Cancer Research Campaign*

BJOC 01-1956 735-740 20/8/01 3:20 pm Page 737

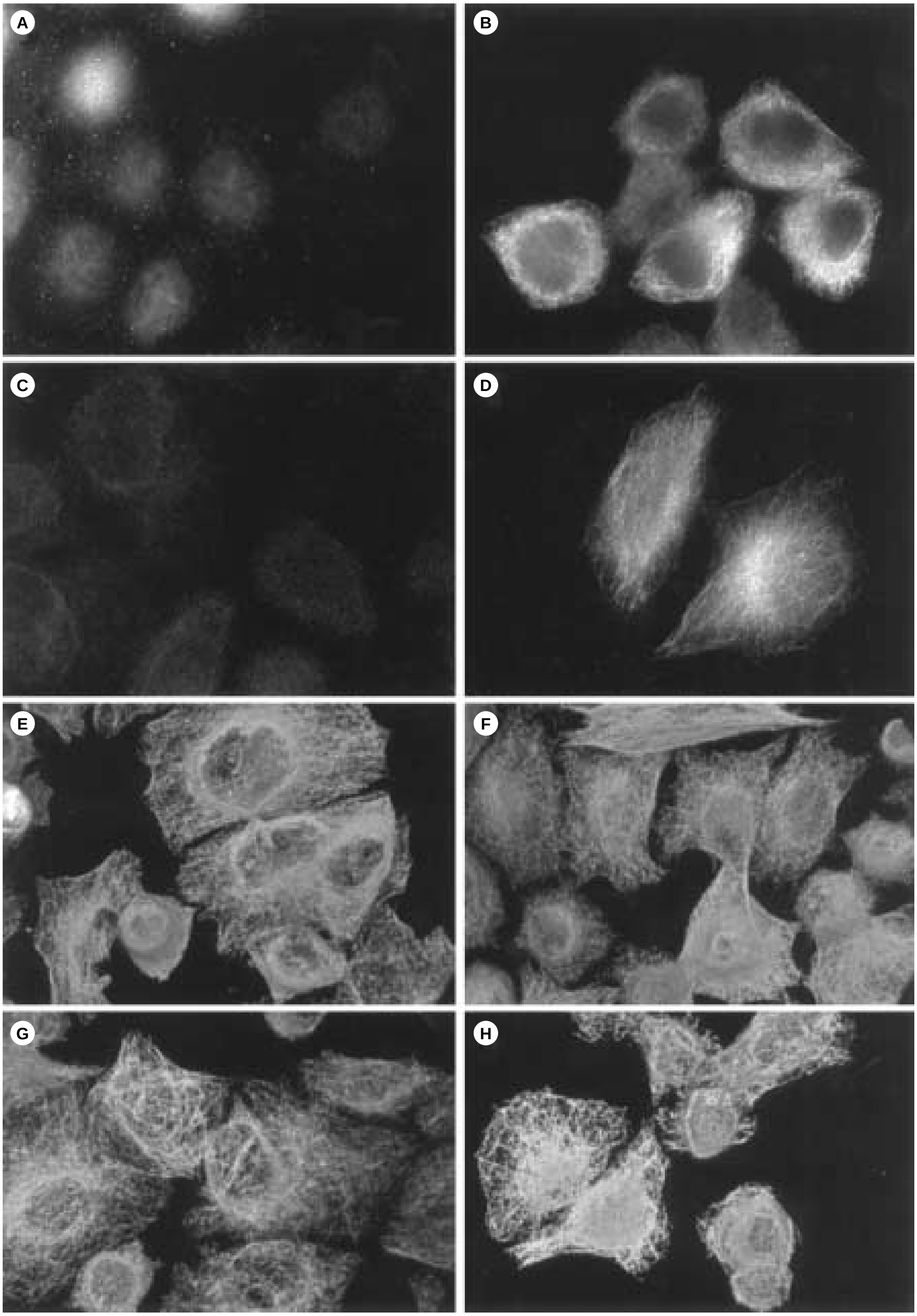

**Figure 1** Immunofluorescent staining of pZeoSV (**A, C, E, G**) and pZeo$\beta_{III}$ (**B, D, F, H**) transfectants. Cells were plated onto coverslips, fixed and stained with the indicated antibodies as described in the Methods section. (**A, B**) $\beta_{III}$ tubulin isotype, (**C, D**) HA-epitope tag, (**E, F**) pan $\beta$ tubulin, and (**G, H**) pan $\alpha$ tubulin

© 2001 Cancer Research Campaign 

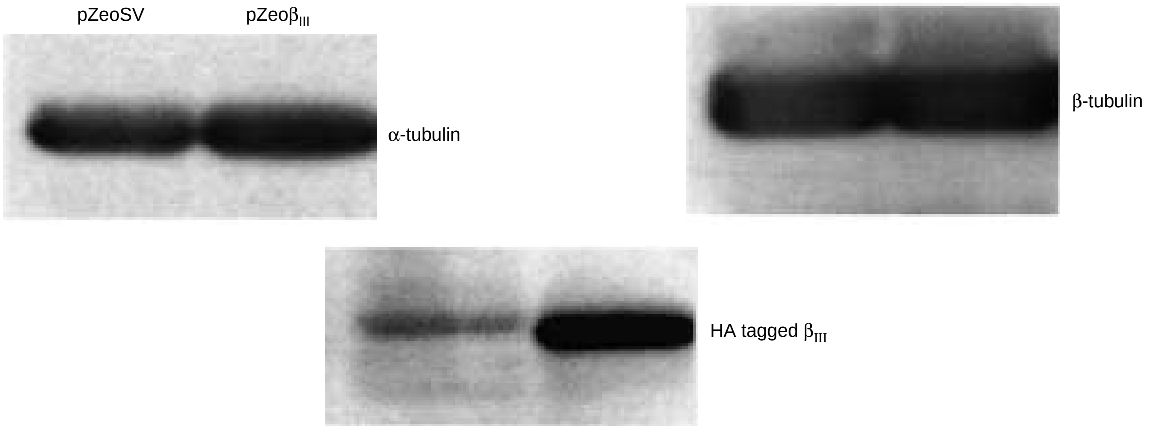

**Figure 2** Western blot analysis of pZeoSV and pZeo$\beta_{III}$ transfectants. Cell lysates were prepared, run on 8% polyacrylamide gels, transferred onto membranes and probed with the appropriate antibodies as described in the Methods section

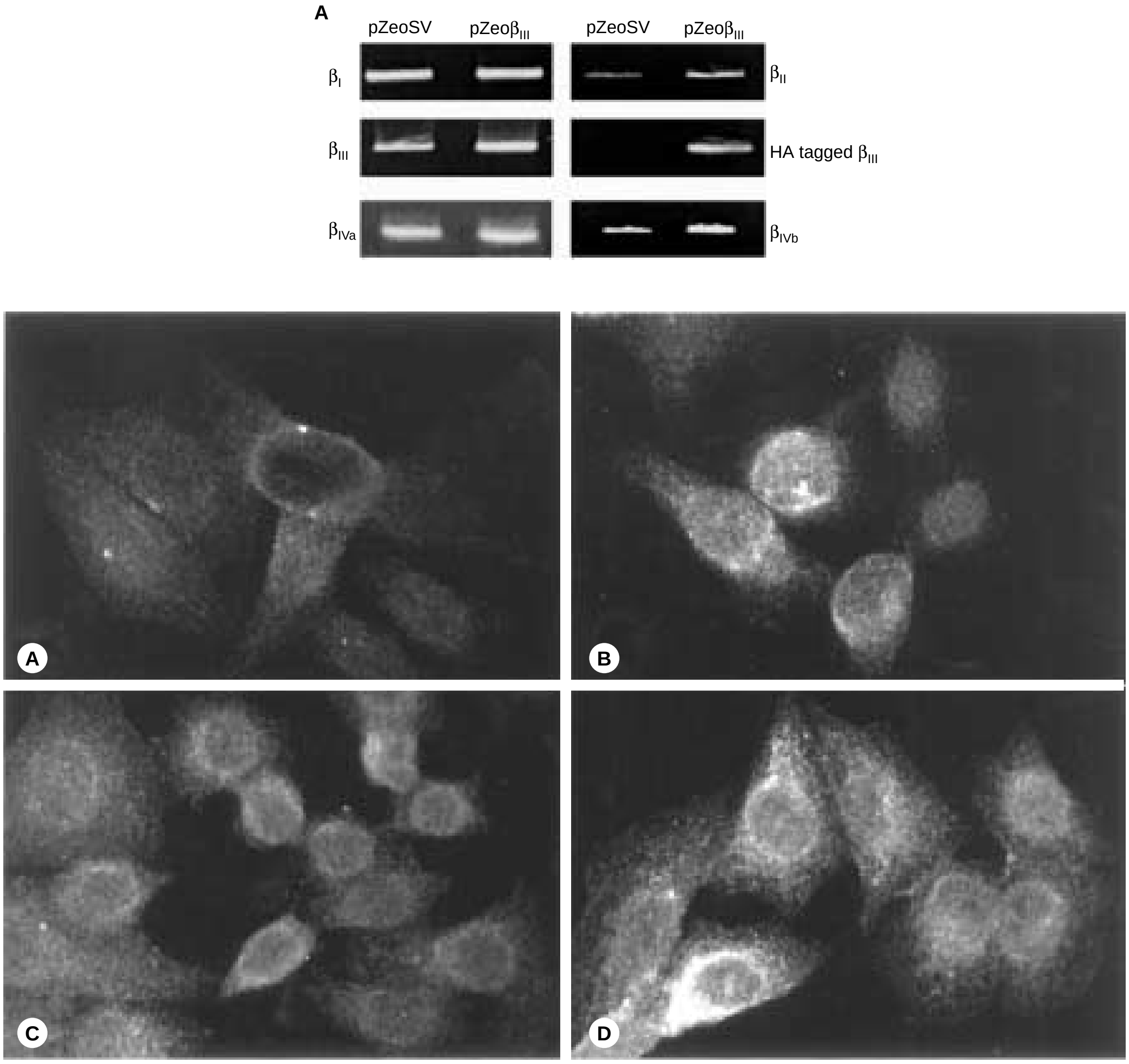

**Figure 3** **(A)** RT-PCR analysis of $\beta$ tubulin transcripts from vector transfected and $\beta_{III}$ transfected cells. RNA was isolated from both cell lines, RT-PCR was performed with primers for individual $\beta$ tubulin isotypes and analysed by agarose gel electrophoresis as described in the Methods. **(B)** Immunofluorescent staining of pZeoSV and pZeo$\beta_{III}$ transfectant cells. Cells were plated onto coverslips, fixed and stained with antibodies against $\beta_{II}$ (**A, B**) and $\beta_{IV}$ (**C, D**) isotypes as described in Methods

 *© 2001 Cancer Research Campaign*

BJOC 01-1956 735-740 20/8/01 3:20 pm Page 739

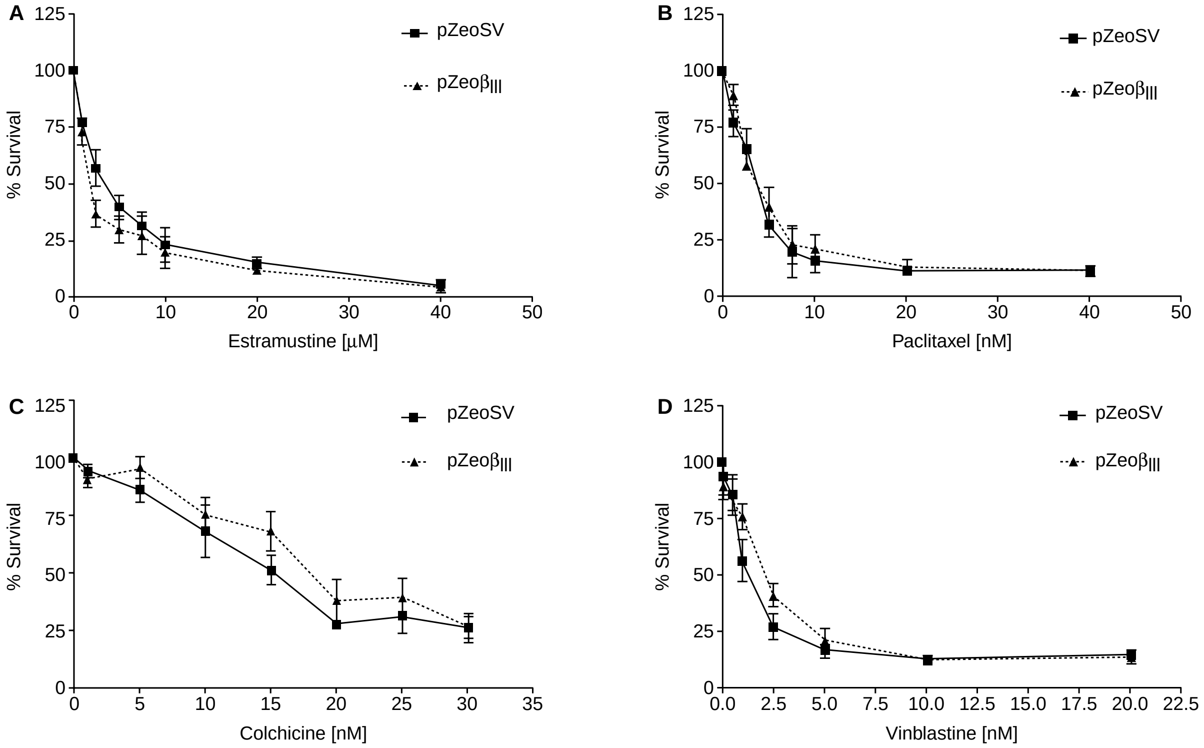

**Figure 4** Dose response curves of pZeoSV and pZeo$\beta_{III}$ cell lines. Cells were plated onto 96-well plates, allowed to attach and treated with indicated doses of antimicrotubule agents for 48 h. Cells were then fixed and stained with sulforhodamine B and cell survivals were determined as described in the methods. (**A**) estramustine, (**B**) paclitaxel, (**C**) colchicine, (**D**) vinblastine

### Coordinated increase of $\beta_{II}$ and $\beta_{IV}$ isotype transcripts in $\beta_{III}$ transfectant cells

RT-PCR analysis of RNA from the transfectant cells has shown that there was a significant increase of $\beta_{III}$ isotype mRNA, as seen by the elevation of overall $\beta_{III}$ levels and HA tagged $\beta_{III}$ levels. (Figure 3A). Quantitation of the image revealed a 3-fold increase of $\beta_{III}$ transcript levels compared to vector transfectants. There was a significant level of HA tagged $\beta_{III}$ transcript compared to no signal in the vector transfectants. $\beta_{I}$ and $\beta_{IVa}$ levels were similar in both cell lines. Interestingly, a collateral increase in the levels of $\beta_{II}$ and $\beta_{IVb}$ transcripts was seen in the $\beta_{III}$ transfected cells. The increases were quantified to be 7-fold and 2-fold for $\beta_{II}$ and $\beta_{IVb}$ transcripts, respectively. Immunofluorescent staining of the cells with corresponding antibodies has confirmed the increase at the protein level (Figure 3B).

### Antimicrotubule drug response of transfectant cells

$\beta_{III}$ transfectant and vector control cell lines were treated with estramustine, paclitaxel, colchicine and vinblastine for 48 h, and dose response curves were determined (Figure 4). Cytotoxicity profiles of both cell lines were similar for each of the antimicrotubule agents tested, with IC50 values of 4 μM, 4nM and 2nM for estramustine, paclitaxel and vinblastine, respectively. For colchicine, the IC50 values were 15 and 17nM for $\beta_{III}$ transfected and vector transfected cells. Cell doubling times for $\beta_{III}$ transfectant and vector control cell lines were found to be very similar, ~22 h indicating that transfection of $\beta_{III}$ into cells did not alter cell cycle times.

### DISCUSSION

Several in vitro studies have demonstrated that the β tubulin isotype composition of microtubules determines the differences in their functions including dynamics, assembly properties and sensitivity to antimicrotubule agents (Banerjee et al, 1990, 1992; Benerjee and Luduena, 1992; Lu and Luduena, 1993, 1994; Panda et al, 1994, 1997; Derry et al, 1997). Because antimicrotubule agents are widely used in cancer therapy, tubulin isotype composition of tumour cells may be an important factor in the success of therapy. Estramustine, vinblastine and paclitaxel have been shown to suppress microtubule dynamics at very low drug concentrations (Jordan et al, 1993, 1996; Dhamodharan et al, 1995; Panda et al, 1997). Work by Lu and Luduena (1993) demonstrated that microtubules lacking the $\beta_{III}$ isotype polymerized at a faster rate in the presence of paclitaxel than the microtubules from unfractioned tubulin. Derry et al (1997) have shown that suppression of microtubule dynamics by paclitaxel varies according to β tubulin isotype content, with $\alpha\beta_{III}$ microtubules and $\alpha\beta_{IV}$ microtubules being approximately 7-fold less sensitive to the drug than the microtubules from unfractionated tubulin.

Work from many laboratories including our own has shown that cancer cell lines made resistant to antimicrotubule agents have altered tubulin isotype composition. In addition, Kavallaris et al (1999) have shown that antisense oligonucleotides to $\beta_{III}$ isotype sensitized paclitaxel resistant lung cancer cells to the drug. However, the data presented in this paper demonstrate that transfection and 3-fold over-expression of $\beta_{III}$ isotype in cells does not confer resistance to estramustine or paclitaxel. Increases of β tubulin isotypes seen in drug resistant cell lines might be due to the

© 2001 Cancer Research Campaign 

selection with antimicrotubule agents. Another reason for the lack of antimicrotubule drug resistance of $\beta_{III}$ transfectant cells might be the collateral increases of $\beta_{II}$ and $\beta_{IVb}$ levels. Cellular β tubulin levels are under autoregulation through cotranslational degradation of mRNAs (Theodorakis and Cleveland, 1992; Bachurski et al, 1994). In addition, Gonzalez-Garay and Cabral (1995) have shown that over-expression of $\beta_I$ tubulin in Chinese hamster ovary cells resulted in coordinate increase of α tubulin levels. Based on the collateral increases of $\beta_{II}$ and $\beta_{IVb}$ levels in our $\beta_{III}$ transfectant cells, it is possible to conceive that cells compensate for over-expression of $\beta_{III}$ by autoregulation of these isotypes.

Tubulin isotypes differ significantly from each other in their assembly properties (Lu and Luduena, 1994). Microtubules from $\alpha\beta_{II}$ and $\alpha\beta_{IV}$ assembled much faster than unfractionated tubulin. $\alpha\beta_{III}$ dimers assembled at a very slow rate and had the highest critical concentration. However, in vitro analysis by Panda et al (1994) has shown that microtubules assembled from $\alpha\beta_{III}$ dimers were more dynamic than the microtubules from $\alpha\beta_{II}$ and $\alpha\beta_{IV}$ isotypes. They further demonstrated that addition of $\alpha\beta_{II}$ dimers to $\alpha\beta_{III}$ dimers suppressed microtubule dynamics. In addition, colchicine binding characteristics of $\alpha\beta_{III}$ dimers were significantly different from $\alpha\beta_{II}$ and $\alpha\beta_{IV}$ dimers, with apparent on-rate constants of 132 ± 5, 30 ± 2 and 236 ± 7 $M^{-1}s^{-1}$ for $\alpha\beta_{II}$, $\alpha\beta_{III}$ and $\alpha\beta_{IV}$ dimers, respectively (Banerjee and Luduena, 1992). The data presented in this paper together with existing in vitro data support the hypothesis that cells might modulate their microtubule functions by altering tubulin isotype composition. The lack of antimicrotubule resistance seen in our $\beta_{III}$ transfectants, $\beta_{IVa}$ transfectants (Wu et al, 1999) and the rodent $\beta_I$, $\beta_{II}$ or $\beta_{IVb}$ transfectants (Blade et al, 1999) altogether point to the conclusion that the β tubulin regulation is highly complex. When cells are transfected with an individual β tubulin isotype, cellular compensatory mechanisms come into play, including altered expression of endogenous β tubulin isotypes, which may alter the effects of the transfected isotype.

### ACKNOWLEDGEMENTS

We wish to thank Jonathan Boyd for his help with microscopy and imaging. We also thank Pat Kraus for her assistance in typing the manuscript.

### REFERENCES

Bachurski CJ, Theodorakis NG, Coulson RMR and Cleveland DW (1994) An amino-terminal tetrapeptide specifies contranslational degradation of β-tubulin and not α-tubulin in mRNAs. *Mol Cell Biol* **14**: 4076–4086

Banerjee A, Roach MC, Trcka P and Luduena RF (1990) Increased microtubule assembly in bovine brain tubulin lacking the type III isotype of β-tubulin. *J Biol Chem* **265**: 1794–1799

Banerjee A, Roach MC, Trcka P and Luduena RF (1992) Preparation of a monoclonal antibody specific for the class IV isotype of β-tubulin. *J Biol Chem* **267**: 5625–5630

Banerjee A and Luduena RF (1992) Kinetics of colchicine binding to purified β-tubulin isotypes from bovine brain. *J Biol Chem* **267**: 13335–13339

Blade K, Menick DR and Cabral F (1999) Overexpression of class I, II or IVb β-tubulin isotypes in CHO cells is insufficient to confer resistance to paclitaxel. *J Cell Science* **112**: 2213–2221

Cabral F, Brady RC and Schibler MJ (1986) A mechanism of cellular resistance to drugs that interfere with microtubule assembly. *Ann NY Acad Sci* **466**: 745–756

Derry B, Wilson L, Khan IA, Luduena RF and Jordan MA (1997) Taxol differentially modulates the dynamics of microtubules assembled from unfractionated and purified β-tubulin isotypes. *Biochemistry* **36**: 3554–3562

Dhamodharan R, Jordan MA, Thrower D, Wilson L and Wadsworth P (1995) Vinblastine suppresses dynamics of individual microtubules in living interphase cells. *Mol Biol Cell* **6**: 1215–1229

Giannakakou P, Sackett DL, Kang Y-K, Zhan Z, Buters JTM, Fojo T and Poruchynsky MS (1997) Paclitgaxel-resistant human ovarian cancer cells have mutant β-tubulins that exhibit impaired paclitaxel-driven polymerization. *J Biol Chem* **272**: 17118–17125

Gonzalez-Garay ML and Cabral F (1995) Overexpression of an epitope-tagged β-tubulin in Chinese hamster ovary cells causes an increase in endogenous α-tubulin synthesis. *Cell Motility and the Cytoskeleton* **31**: 259–272

Haber M, Burkhart CA, Regl DL, Madafiglio J, Norris MD and Horwitz SB (1995) Altered expression of Mβ2, the class II β-tubulin isotype, in a murine J774.2 cell line with a high level of taxol resistance. *J Biol Chem* **270**: 31269–31275

Jordan MA, Toso RJ, Thrower D and Wilson L (1993) Mechanism of mitotic block and inhibition of cell proliferation by taxol at low concentrations. *Proc Natl Acad Sci USA* **90**: 9552–9556

Jordan MA, Wendell K, Gardiner S, Derry WB, Copp H and Wilson L (1996) Mitotic block induced in HeLa cells by low concentrations of paclitaxel (taxol) results in abnormal mitotic exit and apoptotic cell death. *Cancer Res* **56**: 816–825

Kavallaris M, Kuo DY-S, Burkhart CA, Regl DL, Norris MD, Haber M and Horwitz SB (1997) Taxol-resistant epithelial ovarian tumors are associated with altered expression of specific β-tubulin isotypes. *J Clin Invest* **100**: 1282–1293

Kavallaris M, Burkhart CA and Horwitz SB (1999) Antisense oligonucleotides to class III β-tubulin sensitize drug-resistant cells to taxol. *Br J Cancer* **80**: 1020–1025

Lu Q and Luduena RF (1993) Removal of $\beta_{III}$ isotype enhances taxol induced microtubule assembly. *Cell Struct and Function* **18**: 173–182

Lu Q and Luduena RF (1994) *In vitro* analysis of microtubule assembly of isotypically pure tubulin dimers. *J Biol Chem* **269**: 2041–2047

Minotti AM, Barlow SB and Cabral F (1991) Resistance to antimitotic drugs in Chinese hamster ovary cells correlates with changes in the level of polymerized tubulin. *J Biol Chem* **102**: 1522–1531

Monzo M, Rosell R, Sanchez JJ, Lee JS, O'Brate A, Gonzalez-Larriba JL, Alberola V, Lorenzo JC, Nunez L, Ro JY and Martin C (1999) Paclitaxel resistance in non-small-cell lung cancer associated with beta-tubulin gene mutations. *J Clin Oncol* **17**: 1786–1793

Panda D, Miller HP, Benerjee A, Luduena RF and Wilson L (1994) Microtubule dynamics *in vitro* are regulated by the tubulin isotype composition. *Proc Natl Acad Sci USA* **91**: 11358–11362

Panda D, Miller HP, Islam K and Wilson L (1997) Stabilization of microtubule dynamics by estramustine by binding to a novel site in tubulin: a possible mechanistic basis for its antitumor action. *Proc Natl Acad Sci USA* **94**: 10560–10564

Ranganathan S, Dexter DW, Benetatos CA, Chapman A, Tew KD and Hudes GR (1996) Increase of $\beta_{III}$- and $\beta_{IVa}$-tubulin isotopes in human prostate carcinoma cells as a result of estramustine resistance. *Cancer Res* **56**: 2584–2589

Ranganathan S, Benetatos CA, Colarusso PJ, Dexter DW and Hudes GR (1998a) Altered β-tubulin isotype expression in paclitaxel-resistant human prostate carcinoma cells. *Br J Cancer* **77**: 562–566

Ranganathan S, Dexter DW, Benetatos CA and Hudes GR (1998b) Cloning and sequencing of human $\beta_{III}$-tubulin cDNA: induction of $\beta_{III}$ isotype in human prostate carcinoma cells by acute exposure to antimicrotubule agents. *Biochim Biophys Acta* **1395**: 237–245

Ross HJ and Antoniono RJ (1999) Treatment with paclitaxel plus cyclosporin A alters β-tubulin isotype expression in lung and other carcinoma cells *in vitro*. *Proc Am Assoc Cancer Res* **40**: 1245A

Schibler M and Cabral F (1986) Taxol-dependent mutants of Chinese hamster ovary cells with alterations in α- and β-tubulin. *J Cell Biol* **102**: 1522–1531

Theodorakis NG and Cleveland DW (1992) Physical evidence for cotranslational regulation of β-tubulin mRNA degradation. *Mol Cell Biol* **12**: 791–799

Wiesen KM and Horwitz SB (2000) Isolation and characterization of a p-glycoprotein independent taxol-resistant MDA-MB-231 breast carcinoma cell line. *Proc Am Assoc Cancer Res* **141**: 901A

Wu X, Wang Y, Duran GE and Sikic BI (1998) Cloning of the human 5β (class IVa) tubulin gene and expression by a tet-off regulated system. *Proc Am Assoc Cancer Res* **39**

*© 2001 Cancer Research Campaign*
